# Complete mitochondrial genome and the phylogenetic position of a new species, *Johnius taiwanensis* (Perciformes: Sciaenidae) from Chinese waters

**DOI:** 10.1080/23802359.2020.1718563

**Published:** 2020-01-27

**Authors:** Bai-an Lin, Chang-Chang Guo, Lü-Ping Fang, Wei-Di Yang, Min Liu

**Affiliations:** College of Ocean and Earth Sciences, Xiamen University, Xiamen City, Fujian Province, China

**Keywords:** New *Johnius* species, Bayesian tree, mitogenomes, phylogenetic relationship, Sciaenidae

## Abstract

In this study, the complete mitogenome of a new species, *Johnius taiwanensis* (Chao et al. 2019) was obtained. Its mitogenome is 18,451 bp in length, consisting of 37 genes with the typical gene order and direction of transcription in vertebrates. Gene rearrangement was found in *J. taiwanensis*. The overall nucleotide composition is: 24.2% A; 18.0% C; 21.1% G, and 36.7% T. Sizes of the 22 tRNA genes range from 66 to 75 bp. Two start codons (ATG and GTG) and three stop codons (TAG, AGA and TAA/TA/T) were detected in 13 protein-coding genes. In the Bayesian tree based on the complete mitogenomes of 26 species (including *J. taiwanensis*) from the family Sciaenidae, all nodes were strongly supported. The result shows that *J. taiwanensis* was placed as sister to the Trewavas croaker *J. trewavasae* of the same genus. The mechanism of gene rearrangement in the genus *Johnius* merits further investigation.

The family Sciaenidae (Perciformes) is commonly known as croakers and drums. As a commercially important group of fishes, the family comprises approximately 283 species in 67 genera in the world (Nelson et al. 2016). The genus *Johnius* consists of 34 small- to medium-sized species endemic to Indo-West Pacific, usually having a small mouth, sub-terminal to inferior positioned. The body shape and pigmentation are also similar in many *Johnius* species, which has made the identification quite confused in literature. The newly described species, *Johnius taiwanensis* is commonly found along both sides of the Taiwan Strait, from Zhejiang to Guangdong and Hong Kong on the west side, and from west coast of Taiwan on the east side (Chao et al. 2019). In this study, we presented the complete mitochondrial genome of *J. taiwanensis* and analyzed its phylogenetic relationship based on another 26 available mitogenomes in Sciaenidae using two in the family Polynemidae as an outgroup.

One specimen of *J. taiwanensis* (FJDS20160317) was collected by hook-and-line in the coastal water of Dongshan County, Fujian Province, China. The protocol and data analysis methods were according to Li et al. (2015). The complete mitochondrial genome of *J. taiwanensis* is 18,451 bp in length (GenBank accession number: MG917694) with the typical gene order and transcriptional direction in vertebrates and with gene rearrangement feature.

*Johnius taiwanensis* contains 2 rRNA genes, 22 tRNA genes, 13 protein-coding genes, and 5 non-coding regions. The 5 major non-coding regions include one control region and 5 noncoding regions named as NC1–NC5. The overall nucleotide composition is as follows: 24.2% A; 18.0% C; 21.1% G, and 36.7% T. The 12S (958 bp) and 16S (1,701 bp) rRNA genes are located between two non-coding region (NC3 and NC5), separated by a 72 bp noncoding region (NC4). In the 13 protein-coding genes, two start codons (ATG and GTG) were detected. Three stop codons (TAG, AGG, and TAA/TA/T) were found; ND1 and ND5 were terminated by the TAG codon, COX1 by the AGA gene, and the other 10 protein-coding genes by either the TAA or incomplete TA– or T–– codon that may form the complete termination signal UAA via post-transcriptional polyadenylation (Ojala et al. 1981). The lengths of 22 tRNA genes range from 66 to 75 bp without any inserted sequences identified as the putative origin of L-strand replication (OL). The control region was 1320 bp in length with high A + T (65.8%) and low G + C (34.2%) composition, located between the tRNA-*Phe* and tRNA-*Val* genes. The NC1 (571 bp) was located between tRNA-*Thr* and tRNA-*Pro* genes. The NC2 (392 bp) between tRNA-*Pro* genes and tRNA-*Val* genes has five repeats with the consensus sequence of 5′-ATAATAGCTTGTAATTATAA-3′. The NC3 (127 bp) was located between tRNA-*Val* and 12S rRNA genes. The NC4 (72 bp) was located between 12S rRNA and 16S rRNA genes. The NC5 (230 bp) was located between 16S rRNA and tRNA-*Phe* genes. The NC1–NC5 had no features of the control region and showed no homology to the other regions of the same mtDNA or to the sequence data in the public database.

The complete mitogenomes of 26 species from Sciaenidae (including *J. taiwanensis* in this study) together with *Eleutheronema tetradactylum* and *Polydactylus sextarius* from Polynemidae were used to assess the phylogenetic relationship of *J. taiwanensis*. The phylogenetic tree was constructed with the partitioned Bayesian method based on the dataset combined by three partitions (the alignments of the 1, 2 codon positions of 12 H-strand encoded protein-coding genes together with 12S and 16S rRNA genes) under the GRT + G + I model (Ronquist and Huelsenbeck 2003). The phylogenetic tree showed that all nodes were strongly supported with a high value of posterior probability (Figure 1). The result showed that *J. taiwanensis* was placed as a sister to *J. trewavasae*, and the relation with other *Johnius* species was consistent to the croaker phylogenetic results using combined mitochondrial and nuclear genomes (Lo et al. 2015). The mechanism of gene rearrangement in the genus *Johnius* merits further investigation.

**Figure 1.**
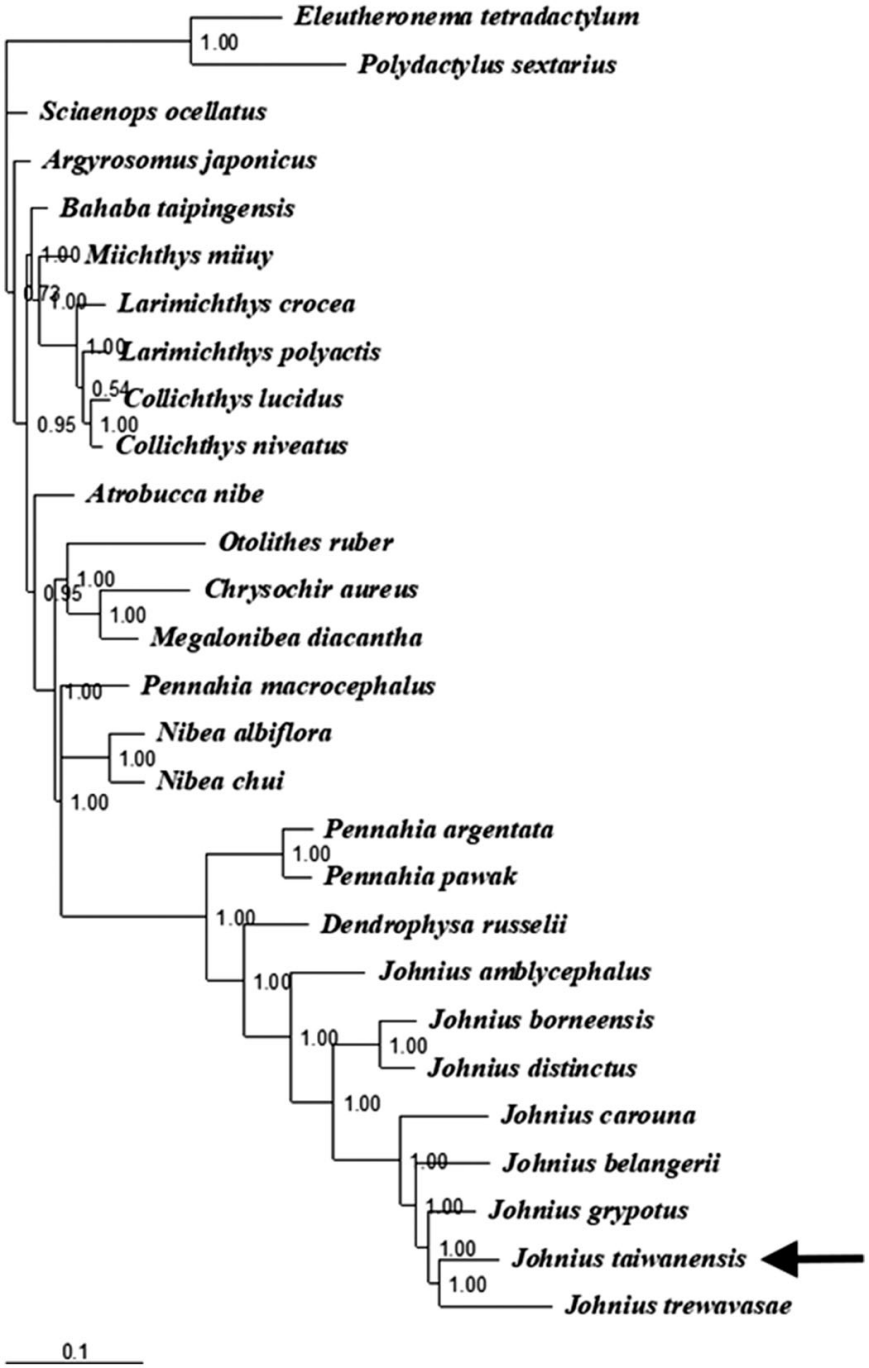
Phylogenetic position of *Johnius taiwanensis* (MG 917694). *Eleutheronema tetradactylum* (KC878730) and *Polydactylus sextarius* (NC_027088) from the family Polynemidae were selected as the outgroup. The other 21 species from the family Sciaenidae are: *Argyrosomus japonicus* (KT184692), *Atrobucca nibe* (MF004314), *Bahaba taipingensis* (NC_018347), *Chrysochir aureus* (MF004313), *Collichthys lucidus* (JN857362), *Collichthys niveatus* (JN678726), *Dendrophysa russelii* (JQ728562), *Johnius carouna* (MF004312), *Johnius distinctus* (MF083699), *Johnius grypotus* (KC491206), *Larimichthys crocea* (NC_011710), *Larimichthys polyactis* (GU586227), *Megalonibea diacantha* (KM257722), *Miichthys miiuy* (NC_014351), *Nibea albiflora* (NC_015205), *Nibea chui* (NC_025307), *Otolithes ruber* (KX929060), *Pennahia argentata* (NC_015202), *Pennahia macrocephalus* (KX576460), *Pennahia pawak* (KY978753) and *Sciaenops ocellatus* (NC_016867).

## Sample collection and DNA storage

The specimen of *J. taiwanensis* was collected from the fish market (Xipu Market) of Dongshan County (117°25 N, 23°41E), Fujian Province, China.

The specimen and DNA of *J. taiwanensis* were stored in the Fish Biology Laboratory of Xiamen University, China.
